# Comparative assessment of synthetic time series generation approaches in healthcare: leveraging patient metadata for accurate data synthesis

**DOI:** 10.1186/s12911-024-02427-0

**Published:** 2024-01-30

**Authors:** Imanol Isasa, Mikel Hernandez, Gorka Epelde, Francisco Londoño, Andoni Beristain, Xabat Larrea, Ane Alberdi, Panagiotis Bamidis, Evdokimos Konstantinidis

**Affiliations:** 1 Digital Health and Biomedical Technologies, Vicomtech Foundation, Basque Research and Technology Alliance (BRTA), Donostia-San Sebastián, Spain; 2 Computer Science and Artificial Intelligence Department, Computer Science Faculty, University of the Basque Country (UPV/EHU), Donostia - San Sebastian, Spain; 3 eHealth Group, Biogipuzkoa Health Research Institute, Donostia-San Sebastian, Spain; 4 Biomedical Engineering Department, Mondragon University, Arrasate-Mondragon, Spain; 5 Laboratory of Medical Physics and Digital Innovation, School of Medicine, Aristotle University of Thessaloniki, Thessaloniki, Greece; 6 European Network of Living Labs (ENoLL), Brussels, Belgium

**Keywords:** Time series, Synthetic data, Privacy-preserving data sharing, Health data

## Abstract

**Background:**

Synthetic data is an emerging approach for addressing legal and regulatory concerns in biomedical research that deals with personal and clinical data, whether as a single tool or through its combination with other privacy enhancing technologies. Generating uncompromised synthetic data could significantly benefit external researchers performing secondary analyses by providing unlimited access to information while fulfilling pertinent regulations. However, the original data to be synthesized (e.g., data acquired in Living Labs) may consist of subjects’ metadata (static) and a longitudinal component (set of time-dependent measurements), making it challenging to produce coherent synthetic counterparts.

**Methods:**

Three synthetic time series generation approaches were defined and compared in this work: only generating the metadata and coupling it with the real time series from the original data (A1), generating both metadata and time series separately to join them afterwards (A2), and jointly generating both metadata and time series (A3). The comparative assessment of the three approaches was carried out using two different synthetic data generation models: the Wasserstein GAN with Gradient Penalty (WGAN-GP) and the DöppelGANger (DGAN). The experiments were performed with three different healthcare-related longitudinal datasets: Treadmill Maximal Effort Test (TMET) measurements from the University of Malaga (1), a hypotension subset derived from the MIMIC-III v1.4 database (2), and a lifelogging dataset named PMData (3).

**Results:**

Three pivotal dimensions were assessed on the generated synthetic data: resemblance to the original data (1), utility (2), and privacy level (3). The optimal approach fluctuates based on the assessed dimension and metric.

**Conclusion:**

The initial characteristics of the datasets to be synthesized play a crucial role in determining the best approach. Coupling synthetic metadata with real time series (A1), as well as jointly generating synthetic time series and metadata (A3), are both competitive methods, while separately generating time series and metadata (A2) appears to perform more poorly overall.

## Background

Amidst the information era in which the digitalization of healthcare applications is being streamlined, privacy is evolving towards an increasingly precious asset. Data protection regulations, such as the GDPR (General Data Protection Regulation) in Europe or the HIPAA (Health Insurance Portability and Accountability Act) in the USA, regulate the processing of Personally Identifiable Information (PII) by, for example, defining the rights of the data subjects, the responsibilities of data processing agents, or various codes of conduct [1]. However, attacks against theoretically secure private datasets are becoming increasingly sophisticated and have managed to break security barriers down [2, 3], which obliges data collectors, controllers, and processors, to implement harsher privacy safeguards.

A relatively novel and mainstream technology to help data leakage risks is Synthetic Data Generation (SDG). This technology consists of creating artificial versions of real data (RD) using generative models, statistical models or models based on expert knowledge that can learn underlying variable distributions and multivariate correlations [4]. Once an SDG model is trained, as many data records as wanted can be synthesized, following the original patterns from the real dataset. However, even if SDG techniques offer certain privacy guarantees, current literature on the topic is evolving towards determining whether the generated synthetic data (SD) is still considered personal data or not [5].

Among the different sectors in which SDG can be used, healthcare is critical in terms of sensitive information, and thus one of the most compromised information sources that needs to be controlled and monitored [6]. In this context, it is common the data to have two components, the first one being classified as subjects’ metadata (the static component, which does not change in time) and the second one being the time series or longitudinal part (the dynamic component, measured across a time axis). Up to now, clinical SDG has mostly been focused on generating private and useful tabular static data, while the longitudinal component, and the joint generation of both, have fallen behind. Considering this scenario, it is worth noting the need to ensure the privacy of both mentioned components [7]. The combination of metadata with time series could potentially help re-identification and compromised data breaches, but synthesizing time series brings inherent challenges with it, as temporal correlations must be learnt by data generators apart from the usual intervariable correlations.

Regarding generative models, the first Generative Adversarial Network (GAN) architecture was presented by Goodfellow et al. in 2014 [8]. Since then, several modified versions have been published to improve its performance for specific tasks in a healthcare context [9]. As for synthesizing time series, TimeGAN [10] was the first specific variant that paved the way for newer networks that can also handle the temporality of the data [11]. However, how time series that are linked to metadata are synthesized remains a field for further research, as current models are solely focused on generating time series, or they generate them jointly with the associated metadata, still having a gap on the comparison analysis of these latter approaches, which is the primary focus of the current work.

In 2022, we proposed two different approaches for the Synthetic Time Series Generation (STSG) task [12], where different combinations of real and synthetic metadata and time series are merged to find the best approach for achieving useful time series. In the present work, a more extensive evaluation of both approaches is carried out assessing the resemblance of the SD with respect to the RD (1), how useful the SD is to build downstream applications (2), and how private the generated information is (3). The alignment and balance of these components is still an open problem in the current literature, as there exists a complex relationship between the three. A strong privacy guarantee is related to a higher bias of the SD with respect to the RD, which makes the utility and the resemblance of the former to decrease. In contrast, maintaining a higher data utility results in compromised SD, since personal information could potentially be extracted from it due to the extensive resemblance between SD and RD. Furthermore, the relevance of each component depends on the target application, as there are critical examples that need to address the privacy issue and sacrifice the resemblance and utility of the SD, but it is not always the case. Moreover, the models we evaluated in Isasa et al. [13] were included as a third STSG approach, where the metadata and the time series are jointly generated using a single model. Drawing from our prior studies, this is, to our understanding, the first work that evaluates three distinct variants to generate SD that combines time series and related metadata. A common evaluation procedure is employed to assess the resemblance, utility and privacy of the results, thereby making a significant contribution to the field.

The remainder of this document is organised as follows: the “Methods” section provides a detailed description of how the SD was generated using multiple model and dataset combinations, as well as how the data was coupled and evaluated after the generation step. Next, the results of the assessment are presented, followed by a discussion, as well as a list of limitations that were found across this research. Finally, the overall conclusion of the comparison is presented.

## Methods

This section first describes the datasets utilized for this work and the preprocessing steps needed to prepare the data. Then, three different STSG approaches are presented, followed by the used SDG models. Finally, the evaluation of the generated SD is explained.

### Data sources

To ensure outcome consistency devoid of data reliance, the experiments were conducted using two different datasets sourced from *Physionet* [14] and a third lifelogging dataset named PMData.

First, a dataset consisting of cardiorespiratory measurements acquired during 992 Treadmill Maximal Exercise Tests (TMET) performed by amateur and professional athletes was considered [15, 16]. The experiments comprised longitudinal measurements of Graded Exercise Tests (GETs) as well as subject-related metadata, and they were carried out on a *Powerjog J Series* treadmill to which a *CPX MedGraphics* gas analyzer and a *Mortara* 12-lead ECG device were connected. With those, the Heart Rate (HR) of the subjects was recorded, along with the oxygen consumption (VO_2_), carbon dioxide generation (VCO_2_), and pulmonary ventilation measures (Respiratory Rate, RR; Exhaled Volume, VE). As for the metadata component, the age (M: 27.10, Q1-Q3: 21.10-36.32), sex (5.66:1 male to female ratio), height (M: 175.00, Q1-Q3: 170.00-180.00), and weight (M: 73.00, Q1-Q3: 66.00-80.23) of the subjects were obtained, along with ambient measurements such as air temperature (M: 22.90, Q1-Q3: 20.80–24.40) and humidity (M: 47.00, Q1-Q3: 42.00–54.00). The GETs comprised a warmup period at a 5 km/h pace, and speed increments ranging from 0.5 to 1 km/h were applied until the consumed oxygen volume was saturated. The subjects recovered the effort at the initial velocity of 5 km/h. The research, published by the Exercise Physiology and Human Performance Lab of the University of Malaga, complied with all the relevant national regulations, and was performed following the tenets of the Helsinki Declaration. The study protocols were approved by the Research Ethics Committee of the University of Malaga, and written informed consent was collected from all the participants or their legal representatives.

Second, a hypotension subset was derived from the MIMIC-III v1.4 (Medical Information Mart for Intensive Care) database [17, 18]. The database consists of deidentified health-related data from patients that stayed in the critical care unit of the Beth Israel Deaconess Medical Center, in Boston, between 2001 and 2012. Two different critical care information systems were utilized to record patient information: the *Philips CareVue Clinical Information System* (Philips Healthcare, Andover, MA), and the *iMDsoft Metavision ICU* (iMDsoft, Needham, MA). Using those, diastolic and systolic pressures, fraction of inspired oxygen (FiO_2_), urine outputs, and administered vasopressors were monitored. Moreover, Glasgow Coma Scale (GCS) scores were recorded, and the longitudinal data was merged with consequent gender and ethnicity metadata. The hypotension subset was obtained by selecting adult (> 18 years) patients having a Mean Arterial Pressure (MAP) lower than 65 mmHg [19]. All the data is in line with the data protection requirements in the HIPAA, and the project was approved by the Institutional Review Boards of the Beth Israel Deaconess Medical Center (Boston, MA), and the Massachusetts Institute of Technology (Cambridge, MA).

Third, a lifelogging dataset was incorporated to the analysis, named PMData [20]. *FitBit Versa 2* smartwatch wristbands were carried by 16 (13 men and 3 women) people for five months for data collection. The information was enriched with *PMSys* sports logging application data, as well as a set of responses to a questionnaire that aimed to collect static information (age, gender, height, and whether the subject has a type A or a type B personality). On the other hand, time series that were acquired during the study ranged from weekly recordings to every-five-second measurements. All participants on this study signed an informed consent form, and the research was carried out under the evaluation of the Norwegian Centre for Research Data (NSD) and in accordance with Norwegian and EU data protection laws.

### Data preprocessing

First, the TMET dataset was preprocessed following the steps defined in Larrea et al. [12]. Starting from a 992-subject dataset, 30 of them were directly excluded due to missing values on subject metadata. Regarding the longitudinal component, experiments with more than 30 lacking data points were excluded, while the others were linearly interpolated to avoid missing values. Since 942 samples were found missing for the HR variable and 4,871 samples were lacking for both VO_2_ and VCO_2_ variables, 12 more experiments were excluded from the research. The final TMET dataset consisted of 950 experiments, resulting in 216,600 time stamps overall for a constant sequence length of 228 samples.

Second, the hypotension subset derived from MIMIC-III was generated by directly querying the Google Cloud Platform (GCP) where it is made available. Subjects with missing values in either the systolic or the diastolic pressure measurements were excluded from the final dataset. Assuming missing values on the remaining measurements would entail no alterations on the subjects’ previous state, the time series were imputed using a technique called forward filling. This technique uses the last valid measurement of each variable to determine the values of the missing cells until a new valid measurement is found. The categorical variables on this dataset were label-encoded for the DGAN, as it is a model-specific requirement. One-hot encoding was considered better for the WGAN-GP not to cause misinterpretations to the model. The gender variable was binarized, while the ethnicities were clustered into five classes. Finally, 674 subjects were included in the dataset, each of them consisting of 48 hourly measurements and resulting in a total of 32,352 time stamps.

Last, the PMData-based dataset for the experiment was built by selecting the heart rate variable (a measurement was acquired every five seconds) from the longitudinal information and joining it to the associated metadata (age, height, gender, and personality), starting the preprocessing step with a dataset size of 20,991,392 samples and seven variables. Considering the subjects contained variable length heart rate series, the length of the shortest series was chosen to truncate the remaining ones. The remaining dataset consisted of 10,155,936 samples with a time series length of 634,746 samples per subject. Finally, the generation process of the synthetic time series had to be limited to a length of 50,000 samples due to computational resource limitations.

### Synthetic time series generation approaches

**Fig. 1:**
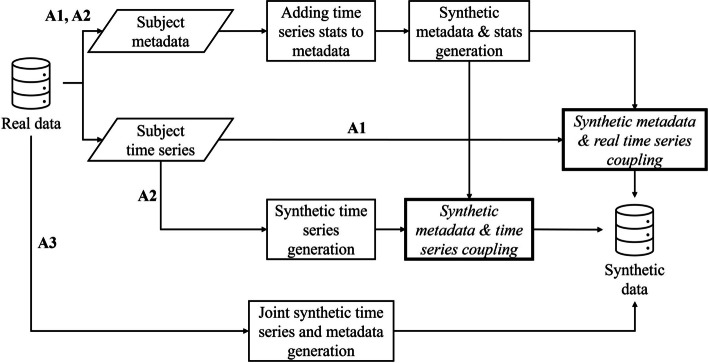
Workflow of the three approaches that were compared in this research. Accentuated boxes refer to the coupling steps of A1 and A2

In this study, three different approaches to generate synthetic time series are presented and compared, namely synthetic metadata generation and real time series coupling or A1, separate synthetic metadata and time series generation and later coupling or A2, and joint synthetic metadata and time series generation, A3. The first two by Larrea et al. [12] were earlier introduced in the manuscript, as well as the third approach considered by Isasa et al. in [13]. In this section, more detailed explanations about the different approaches can be found supported by Fig. 1, which graphically represents the workflow each method follows to generate SD.

The first approach (A1) assumes that no private information is disclosed within real time series data. This approach was inspired by Schiff et al. [21], which is based on enriching synthetic subject metadata with real time series. To do that, meaningful statistical measurements (maximum, minimum, and mean values of each time series) are calculated from the real measurement sequence to be treated as metadata. Next, a synthetic counterpart of the metadata is generated, consisting of synthetic subject metadata and synthetic time series statistical measurements. Finally, the synthetic metadata is coherently coupled to the real time series by pairing the generated values with the real ones so that the distance between them is as small as possible.

As for the second approach (A2), the same meaningful statistical measurements are calculated from the set of real time series. However, in this case, a synthetic set of time series is separately generated. Then, the same coherent coupling is conducted between the synthetic metadata and synthetic time series that were separately generated.

Lastly, the third approach (A3) assumes that subject metadata positively impacts the quality of the generated time series. In this case, subject metadata and time series are generated in a single step.

### Synthetic data generation models

The approaches presented in the previous section were tested using two different SDG models that can incorporate metadata into the time series generation task. Hyperparameter values were experimentally selected for both models and remained unchanged among approximation and dataset combinations.

The Wasserstein GAN with Gradient Penalty (WGAN-GP) was first used with the additional alignment loss feature proposed in the *Health Gym project* [22]. The alignment loss shapes the generator loss function (${\mathcal{L}}_{G}$) by calculating the *L1 norm* of the disparity between intervariable correlation coefficients in the real and synthetic datasets, as defined below:

$${\mathcal{L}}_{G}=-\mathbb{E}\left[D\left(G\left(z\right)\right)\right]+\lambda \sum\limits_{i=1}^{n}\sum\limits_{j=1}^{i-1}{\parallel{\widehat{r}}_{i,j}-{r}_{i,j}\parallel}_{L1}$$

where $\lambda$ is the alignment coefficient, ${\widehat{r}}_{i,j}$ is the correlation coefficient between two synthetically generated variables and ${r}_{i,j}$ is its real counterpart. With this loss function, the generator network gets more penalized if the generated data is not appropriately correlated.

The WGAN-GP was trained for 5,000 epochs using a batch size of 64 samples for the TMET dataset, while for the hypotension subset, a batch size of 32 samples was selected. For the PMData dataset, a batch size of 2 was used due to the low number of subjects. The critic network was updated 5 times more than the generator network at a constant learning rate of 0.001 for all the datasets. Also, the alignment coefficient remained constant at a value of 10.0.

The second generative model used in this research is the DöppelGANger or DGAN [23]. The DGAN first generates the metadata to posteriorly generate time series conditionally on top of it. A Multi-Layer Perceptron (MLP) generates the metadata component, and the output is incorporated into a Recurrent Neural Network (RNN) at each time step, whose task is to generate the longitudinal data. In the DGAN, batch generation is used to generate *S* records per each RNN iteration, a process that aims to capture longer-term effects from the time series. Additionally, a complex discriminator architecture is incorporated into the model, by adding an auxiliary discriminator to focus solely on the metadata. A combined discriminator is responsible for ultimately classifying samples as real or fake. The batch sizes that were used to train the DGAN models were equal to the ones used for the WGAN-GP models. An *S* value of 12 was considered for the TMET dataset, a value of 6 for the hypotension subset, and a value of 127 for the PMData dataset. For these models, a constant learning rate of 0.001 was also utilized for a training process of 5,000 epochs, while the gradient penalty coefficient was set to 10.0 for the DGAN models.

### Synthetic data evaluation

The SD generated using different approaches was evaluated against diverse metrics, quantifying its resemblance to the original data (1), its utility for building downstream applications (2), and the level of privacy of the synthetically generated data (3).

Since generative models produce different data assets each time they are used, a pseudo-cross-validation methodology was incorporated into the evaluation pipeline to eliminate the potential bias due to this aspect. Eight different equally dimensioned synthetic datasets per model and used dataset were generated and used as pseudo-cross-validation folds for this methodology. The results for each evaluation metric and method were combined by calculating the mean value of the different folds. The evaluation methods used in this analysis were mostly based on Hernandez et al. [24], who proposed a collection of objective and universal metrics for evaluating and comparing SDG models and approaches.

The resemblance metrics calculated in this work aim to assess how similar synthetic datasets are with respect to their real counterparts, considering statistical, distributional, and correlational characteristics between them. For this purpose, a multivariate analysis was performed first to check for inter-variable correlations. Numerical variable pairs were evaluated using the *Pearson correlation* coefficient, while categorical variable pairs were examined with the *Cramér’s V value* that was calculated from previous ${\chi }^{2}$-tests. Mixed-type correlations were assessed using the ${R}^{2}$ coefficient of a linear regression of the numeric feature over the categorical one. A final correlation matrix was generated from the SD using the mean value of every fold on the pseudo-cross-validation process. The final matrix was compared to the correlations corresponding to the real dataset using the cosine similarity metric, which is defined as $1-{d}_{cos}$, where a higher value denotes a better correlation preservation.

$${d}_{cos}(x, x') = \frac{\sum _{i=1}^{n} {x}_{i}\cdot{x}_{i}^{'}} {\sum _{i=1}^{n}{x}_{i}\cdot\sum _{i=1}^{n}{x}_{i}^{'}}$$

where both ${x}_{i}$ and ${x}_{i}^{{\prime }}$ refer to the samples to be compared.

Another method to evaluate how SD resembles real data is the Data Labelling Analysis (DLA). This process consists of training several classification models (Random Forests, k-Nearest Neighbors, Decision Trees, Support Vector Machines, and a MLP, in this case) to see if they can distinguish synthetic from real samples, simulating a GAN discriminator. The details on how each classification model was configured can be found in Table 1. The prediction performance was assessed using the Area Under the Receiver Operating Characteristic curve (AUROC), random predictions implying values around 0.5, and the models being unable to distinguish samples from the different sources.

**Table 1:** Classification model parameters

Model	Configuration
RF	Number of trees: 100, split criteria: Gini index
KNN	Number of neighbors: 10
DT	Split criteria: Gini index
SVM	Regularization parameter: 100, maximum epochs: 300, kernel type: linear
MLP	Maximum epochs: 100, hidden layers: (a) 64 neurons and (b) 32 neurons, activation: relu, solver: adam, batch size: 200, learning rate: 0.001

From the proposal of Sajjadi et al. [25], Precision Recall Distribution (PRD) plots were also generated to easily quantify the quality of the model using a single numeric value pair. In their publication, a notion of precision, $\alpha$, and recall, $\beta$, is presented to compare two distributions, the former approximating the quality of the generated samples, and the latter measuring the overlapped proportion between them. Both $\alpha$ and $\beta$ can be merged on a single metric by computing the maximum F-score as shown in the following equation:

$${F}_{\gamma} = \left(1+{\gamma }^{2}\right)\frac{\alpha \cdot \beta }{\left({\gamma}^{2} {\alpha} \right) + \beta}$$

where $\gamma$ is a positive real factor that weighs the recall with regard to the precision. For this analysis, a $\gamma$ value of 8 was empirically selected for the evaluation, weighing the recall higher, although it was not of major importance as a relative analysis was prioritized over an absolute assessment. Moreover, adjustments in the $\gamma$ value would result in an absolute repositioning of the values, with no alteration in the relative differences between approaches nor models.

Data utility refers to the degree to which the SD effectively mimics the original data for analytical or modelling purposes. Regarding that definition, in this work, a Train on Synthetic Test on Real (TSTR) setup was tested against a Train on Real Test on Real (TRTR) one. In these, various Machine Learning (ML) model twins are trained on RD first and SD afterwards, separately. Each twin is tested against an equal real testing set. A regression task was defined for the ML models in both cases, with the VE and GCS variables as the targets on the TMET dataset and the hypotension subset. In this work, each model’s performance was evaluated with the Mean Absolute Error (MAE), and the models trained on SD were compared to the RD counterpart using the cosine similarity metric previously presented. This similarity metric is proportional to the MAE similarity, which gives information about the utility of the synthetic data.

The significance of the observed resemblance and utility metric differences between approaches was assessed using *t*-tests. For a Significance Level (SL) set at 0.05, a *p*-value below it implies the null hypothesis rejection, meaning that differences being tested are statistically significant.

Finally, in a SDG context, privacy can be defined as the extent to which the generated data protects the sensitive information by preventing the membership of real individuals being disclosed in the original dataset. In this analysis, the privacy was evaluated using a Membership Inference Attack (MIA) setup. In this attack, an adversary was considered to possess a proportion of the original data used to train the generative models, ranging from 10 to 50%. Also, SD was thought to be publicly available and, therefore, accessible to the adversary, as it is one of the strengths of SD. For every generated synthetic sample, the cosine distance was calculated with respect to every real sample in the original dataset. A distance value of 0.2 was used as a threshold to consider a synthetic sample similar enough to mark it as one coming from the RD used to train the generative models. As a longitudinal data setup, a single match between the two datasets led to the subject being marked as disclosed.

## Results

In this section, the results of the resemblance, utility, and privacy analyses are presented. In the interest of clarity and simplicity, the models we refer to in this section are subscripted with letters T, M or P, referring to TMET, MIMIC or PMData, which indicate the dataset used in each case to train the model. Also, the *p*-values derived from all the *t*-test calculations that were performed during the analysis can be observed in Tables 2 and 3.

**Table 2:** T-test *p*-values perfomed between approach pairs for the resemblance metrics

Model	Approach comparison	DLA AUROC^a^	Correlation Similarity^a^	Autocorrelation MAE^a^	${\boldsymbol{F}_{\boldsymbol{\gamma}}}$^a^
**DGAN**_**T**_	A1-A2	** ~ **1.00	**3.39 × 10**^**–8**^	**9.33 × 10**^**–4**^	**3.47 × 10**^**–8**^
	A2-A3	7.46 × 10^–1^	**7.25 × 10**^**–12**^	**1.10 × 10**^**–4**^	**1.80 × 10**^**–6**^
	A3-A1	7.90 × 10^–1^	**5.83 × 10**^**–6**^	2.04 × 10^–1^	**1.26 × 10**^**–9**^
**WGAN-GP**_**T**_	A1-A2	8.36 × 10^–1^	**5.56 × 10**^**–11**^	** ~ 0.00**	**2.30 × 10**^**–11**^
	A2-A3	4.44 × 10^–1^	**2.22 × 10**^**–16**^	** ~ 0.00**	**9.11 × 10**^**–12**^
	A3-A1	2.64 × 10^–1^	** ~ 0.00**	** ~ 0.00**	5.37 × 10^–1^
**DGAN**_**M**_	A1-A2	**4.20 × 10**^**–13**^	**9.17 × 10**^**–6**^	5.67 × 10^–2^	**1.82 × 10**^**–10**^
	A2-A3	**4.19 × 10**^**–4**^	5.50 × 10^–2^	** ~ 0.00**	3.15 × 10^–1^
	A3-A1	**8.90 × 10**^**–14**^	**3.34 × 10**^**–7**^	** ~ 0.00**	**8.88 × 10**^**–16**^
**WGAN-GP**_**M**_	A1-A2	**1.36 × 10**^**–6**^	**1.18 × 10**^**–7**^	**1.46 × 10**^**–9**^	**3.42 × 10**^**–3**^
	A2-A3	**5.60 × 10**^**–5**^	**2.20 × 10**^**–11**^	** ~ 0.00**	7.31 × 10^–1^
	A3-A1	**1.01 × 10**^**–9**^	** ~ 0.00**	** ~ 0.00**	**9.92 × 10**^**–12**^
**DGAN**_**P**_	A1-A2	1.00	1.379 × 10^–1^	**4.001 × 10**^**–10**^	**1.811 × 10**^**–6**^
	A2-A3	1.00	**1.806 × 10**^**–6**^	**4.563 × 10**^**–10**^	**3.659 × 10**^**–3**^
	A3-A1	1.00	**2.114 × 10**^**–5**^	**4.911 × 10**^**–12**^	**1.029 × 10**^**–9**^
**WGAN-GP**_**P**_	A1-A2	1.00	**3.718 × 10**^**–3**^	**3.044 × 10**^**–12**^	**8.882 × 10**^**–16**^
	A2-A3	1.00	8.677 × 10^–1^	** ~ 0.00**	**1.222 × 10**^**–7**^
	A3-A1	1.00	**1.510 × 10**^**–4**^	**4.441 × 10**^**–16**^	**4.996 × 10**^**–14**^

^a^Significant results for a $SL=0.05$ are underlined and bold

**Table 3:** T-test *p*-values perfomed between approach pairs for the utility metrics

Model	Approach comparison	TSTR metric^a^
**DGAN**_**T**_	A1-A2	6.46 × 10^−1^
	A2-A3	9.31 × 10^−1^
	A3-A1	7.23 × 10^−2^
**WGAN-GP**_**T**_	A1-A2	2.83 × 10^−1^
	A2-A3	3.65 × 10^−1^
	A3-A1	7.22 × 10^−1^
**DGAN**_**M**_	A1-A2	1.04 × 10^−1^
	A2-A3	5.51 × 10^−1^
	A3-A1	3.08 × 10^−1^
**WGAN-GP**_**M**_	A1-A2	6.10 × 10^−1^
	A2-A3	**3.56 × 10**^**−3**^
	A3-A1	**8.98 × 10**^**−3**^
**DGAN**_**P**_	A1-A2	**2.02 × 10**^**−2**^
	A2-A3	1.10 × 10^−1^
	A3-A1	**6.20 × 10**^**−4**^
**WGAN-GP**_**P**_	A1-A2	**~ 0.00**
	A2-A3	**5.02 × 10**^**−3**^
	A3-A1	**2.33 × 10**^**−4**^

^a^Significant results for a $SL=0.05$ are underlined and bold

Regarding the resemblance, the DGAN_T_ model has resulted in near-perfect precision ($\alpha$: 0.975–0.987) and recall ($\beta$: 0.974–0.994) values on the A1 PRD plot, as well as the DGAN_M_ model ($\alpha$: 0.983–0.990, $\beta$: 0.984–0.994). The DGAN_P_ model matched the recall ($\beta :$ 0.953–0.985) and precision metrics achieved by the previous models to a great extent, even if the latter’s variability between different iterations was high ($\alpha :$ 0.643–0.941). The A2 counterparts of the models have shown a higher deviation inside each pseudo-cross-validation fold, resulting in precision values ranging from 0.918 to 0.970 and recall values ranging from 0.785 to 0.893 for the TMET dataset, precision values from 0.906–0.939 and recall values from 0.705–0.813 for the hypotension subset, and precision values from 0.640–0.877 and recall values between 0.472–0.794 for the PMData dataset. The A3 approach for the DGAN noted further differences between datasets, resulting in a better performance with the TMET dataset ($\alpha$: 0.984–0.987, $\beta$: 0.933–0.950) with respect to the hypotension subset ($\alpha$: 0.966–0.976, $\beta$: 0.720–0.763), and even more with the PMData one ($\alpha$: 0.160–0.297, $\beta$: 0.425–0.704).

Regarding the WGAN-GP_T_ models, recall values from 0.974 to 0.987 and precision values around 0.987 were achieved after the pseudo-cross-validation evaluation of the A1 approach. For A2, the $\beta$ value ranged from 0.903 to 0.934, and $\alpha$ fluctuated between 0.491 and 0.530, while for A3, the former was between 0.979 and 0.986, and the latter was between 0.865 and 0.886. For the WGAN-GP_M_ models, the results on the A1 coupling varied between 0.933 and 0.947 for $\beta$ and between 0.918 and 0.951 for $\alpha$. The A2 method led to worse results for both precision and recall, the former ranging from 0.814 to 0.884 and the latter varying between 0.610 and 0.679. Third, the joint generation of A3 resulted in $\beta$ values between 0.932 and 0. 0.947 and $\alpha$ values between 0.918 and 0.951.

**Fig. 2:**
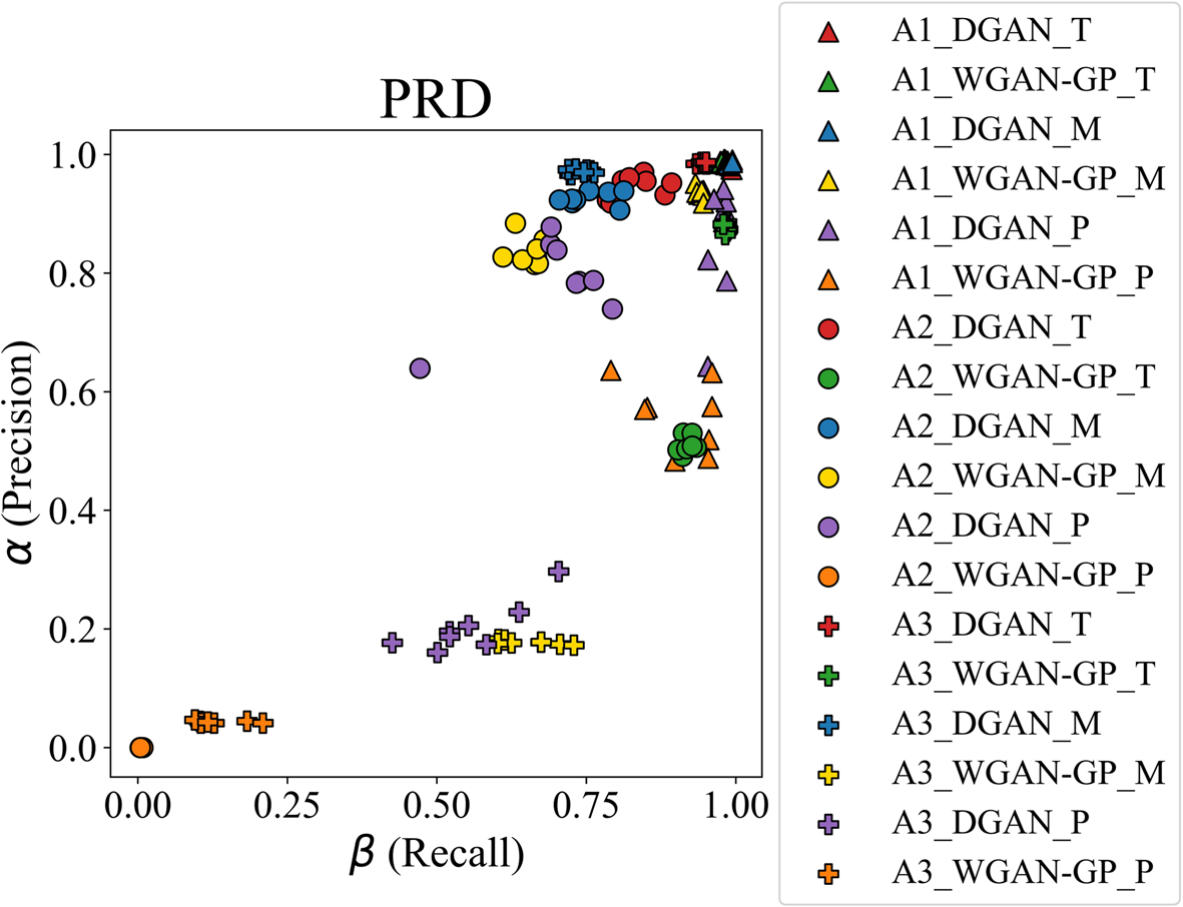
Precision and recall value pairs for each generated dataset. There are eight markers per model and dataset combination. The recall metric is equivalent to the overlapped area between the real and synthetic distributions, while the precision refers to the sample quality

Last, the best results among the WGAN-GP_P_ models were achieved with the A1 approach, varying between an $\alpha$ of 0.483–0.636 and a $\beta$ of 0.791–0.960. A3 was the second best approach for the PMData dataset ($\alpha$: 0.041–0.047, $\beta$: 0.095–0.209), followed by A2 that did not achieve to cover the real distribution nor the quality of the original data ($\alpha$: 0, $\beta$: 0.003–0.008). In Fig. 2 the PRD plot with every pseudo-cross-validation $\alpha$ and $\beta$ value pairs can be observed, and the ${F}_{8}$-scores combining these values are shown in Table 4, where it can be observed that A1 outperforms the other two approaches.

**Table 4:** Resemblance metric results

Generation Method	Model^a^	DLA AUROC	Correlation Similarity	Autocorrelation MAE	${\boldsymbol{F}_{\boldsymbol{\gamma}}}$
**A1**	DGAN_T_	0.902 ± 0.010	0.880 ± 0.010	2.601 ± 0.176	**0.987 ± 0.006**
	WGAN-GP_T_	0.900 ± 0.004	**0.979 ± 0.004**	2.575 ± 0.058	0.980 ± 0.004
	DGAN_M_	**0.744 ± 0.012**	**0.929 ± 0.014**	1.526 ± 0.043	**0.989 ± 0.005**
	WGAN-GP_M_	**0.814 ± 0.016**	**0.942 ± 0.015**	1.527 ± 0.046	**0.942 ± 0.005**
	DGAN_P_	1.000 ± 0.000	0.624 ± 0.091	1.004 ± 0.174	**0.970 ± 0.015**
	WGAN-GP_P_	1.000 ± 0.000	**0.743 ± 0.049**	0.998 ± 0.074	**0.893 ± 0.063**
**A2**	DGAN_T_	0.902 ± 0.008	0.823 ± 0.011	3.190 ± 0.358	0.836 ± 0.039
	WGAN-GP_T_	0.903 ± 0.040	0.951 ± 0.002	**4.160 ± 0.023**	0.908 ± 0.010
	DGAN_M_	0.890 ± 0.011	0.645 ± 0.118	**1.582 ± 0.063**	0.758 ± 0.040
	WGAN-GP_M_	0.898 ± 0.025	0.797 ± 0.039	**1.842 ± 0.045**	0.656 ± 0.230
	DGAN_P_	1.000 ± 0.000	0.550 ± 0.097	**1.944 ± 0.005**	0.699 ± 0.097
	WGAN-GP_P_	1.000 ± 0.000	0.657 ± 0.050	**1.573 ± 0.004**	0.000 ± 0.000
**A3**	DGAN_T_	**0.901 ± 0.003**	**0.906 ± 0.003**	**2.518 ± 0.006**	0.945 ± 0.006
	WGAN-GP_T_	**0.890 ± 0.024**	0.894 ± 0.003	5.789 ± 0.074	**0.981 ± 0.002**
	DGAN_M_	0.871 ± 0.004	0.742 ± 0.057	3.985 ± 0.036	0.742 ± 0.017
	WGAN-GP_M_	0.972 ± 0.027	0.527 ± 0.010	5.840 ± 0.046	0.627 ± 0.044
	DGAN_P_	0.999 ± 0.000	**0.865 ± 0.060**	3.249 ± 0.244	0.541 ± 0.084
	WGAN-GP_P_	1.000 ± 0.000	0.654 ± 0.000	2.082 ± 0.006	0.128 ± 0.037

Values in bold highlight the best-performing approach per trained model and metric

*DLA* Data Labelling Analysis, *AUROC* Area Under the Receiver Operating Characteristic Curve, *MAE* Mean Absolute Error, *DGAN* DöppgelGANger, *WGAN-GP* Wasserstein Generative Adversarial Network with Gradient Penalty

^a^The subscripts on the model column refer to the dataset used for the training step

**Fig. 3:**
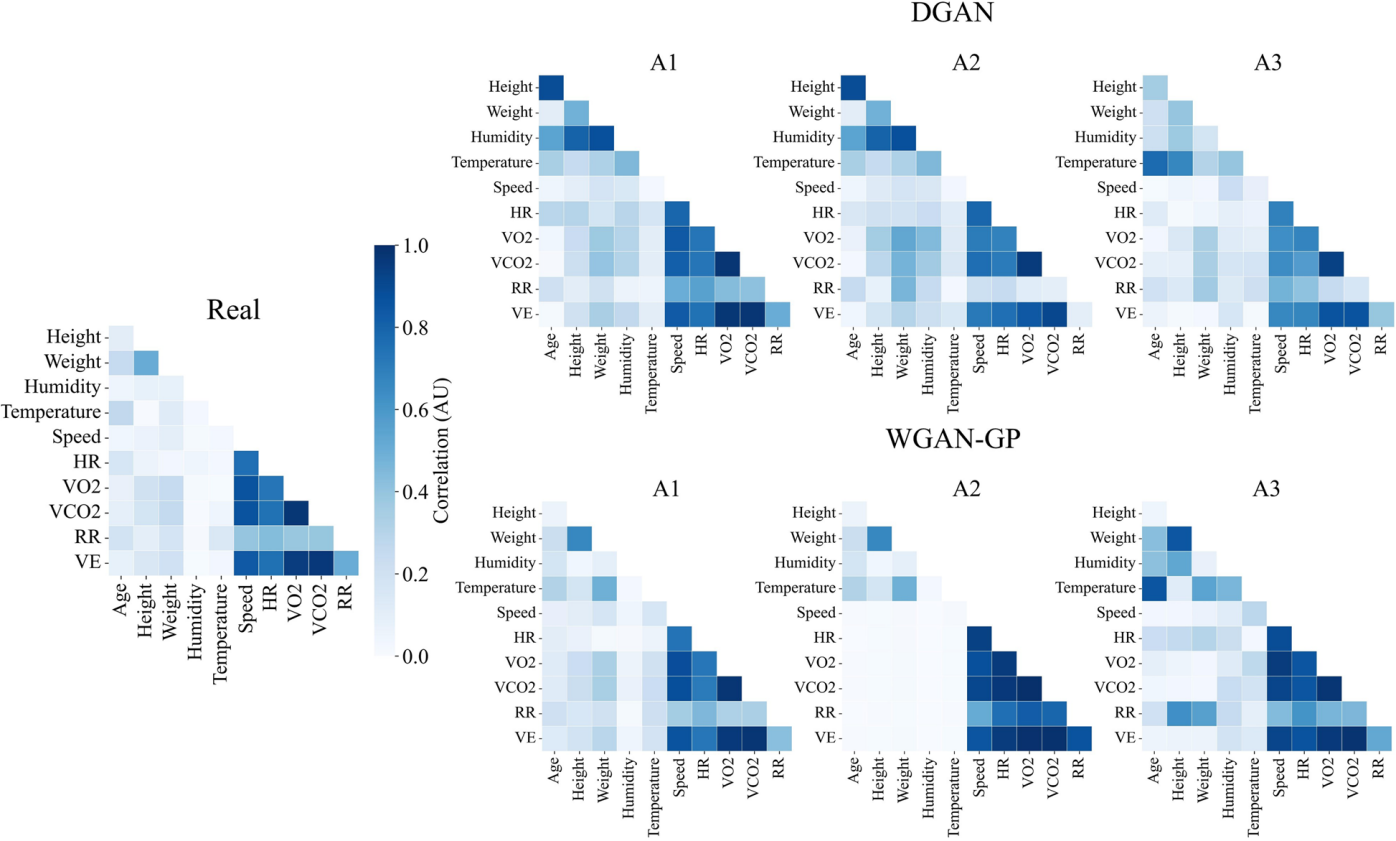
Multivariate correlations of the real (left) and synthetic TMET datasets. The upper row shows the data generated with the DGAN, while the lower row shows the data generated with the WGAN-GP. The three columns on the right refer to the different approaches followed during the synthesis. HR: Heart Rate; VO_2_: Oxygen Volume; VCO_2_: Carbon Dioxide Volume; RR: Respiratory Rate; VE: Exhaled Volume

The multivariate analysis on the DGAN_T_ models has shown that the most realistic correlation matrix was achieved using the A3 approach, as it can be visually appreciated in Fig. 3 and numerically confirmed in Table 4. Regarding the WGAN-GP_T_ model, the best correlation was achieved with the A1 approach, and the worst one was achieved with the A2 approach. Figure 3 shows how the subjects’ metadata in A2 is not correlated to the time series set.

**Fig. 4:**
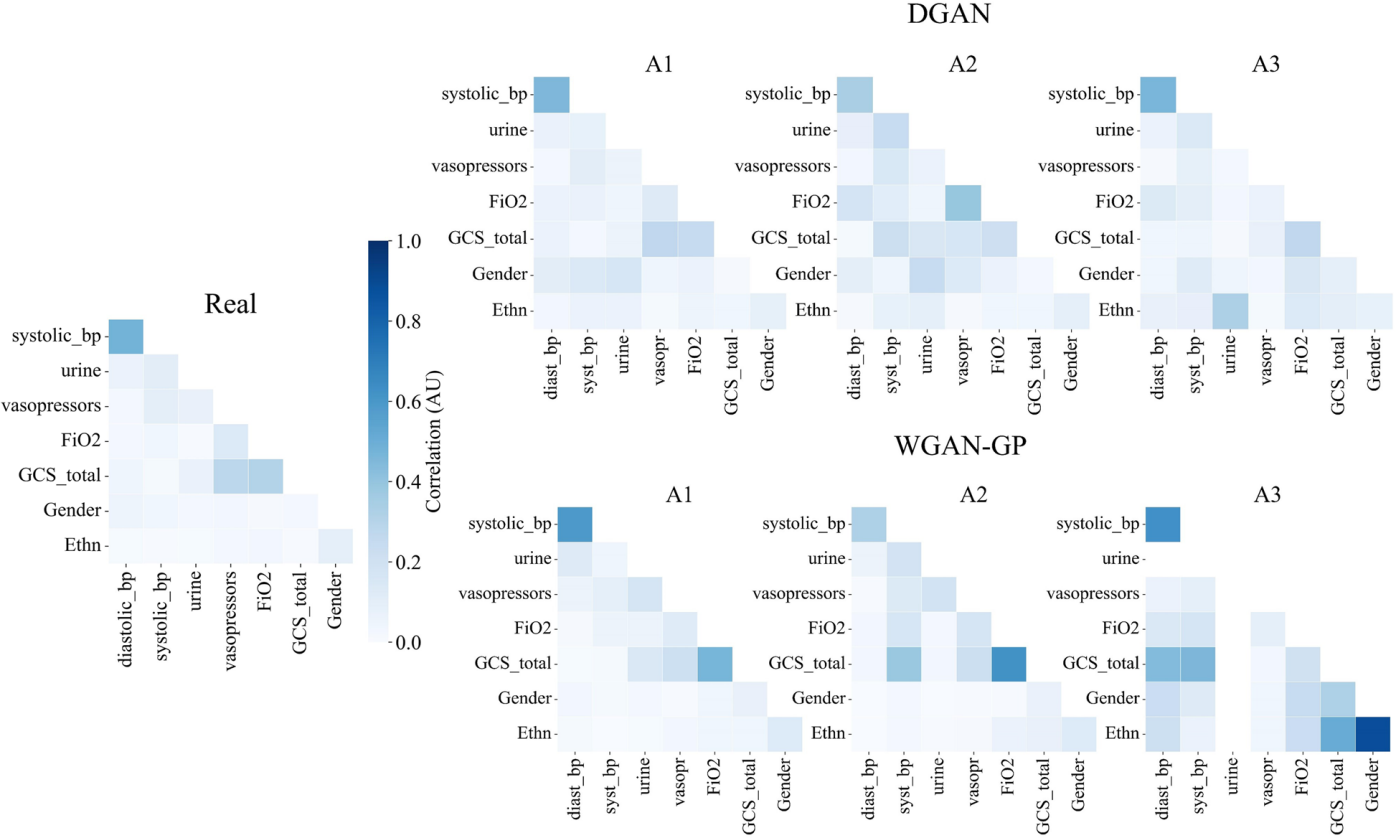
Multivariate correlations of the real (left) and synthetic hypotension datasets. The upper row shows the data generated with the DGAN, while the lower row shows the data generated with the WGAN-GP. The three columns on the right refer to the different approaches followed during the synthesis. GCS: Glasgow Comma Scale. Note that blank values appear for features with a standard deviation equal to zero, which leads to a null value in correlation calculations

As for the DGAN_M_ models, the A1 was the best-performing one, just as it can be observed in Fig. 4 and corroborated in Table 4. The WGAN-GP counterparts on the TMET dataset show worsening intervariable correlations that are proportional to the syntheticity of the data, best performing on A1 and worst performing on A3, just as the WGAN-GP models trained on the hypotension subset.

**Fig. 5:**
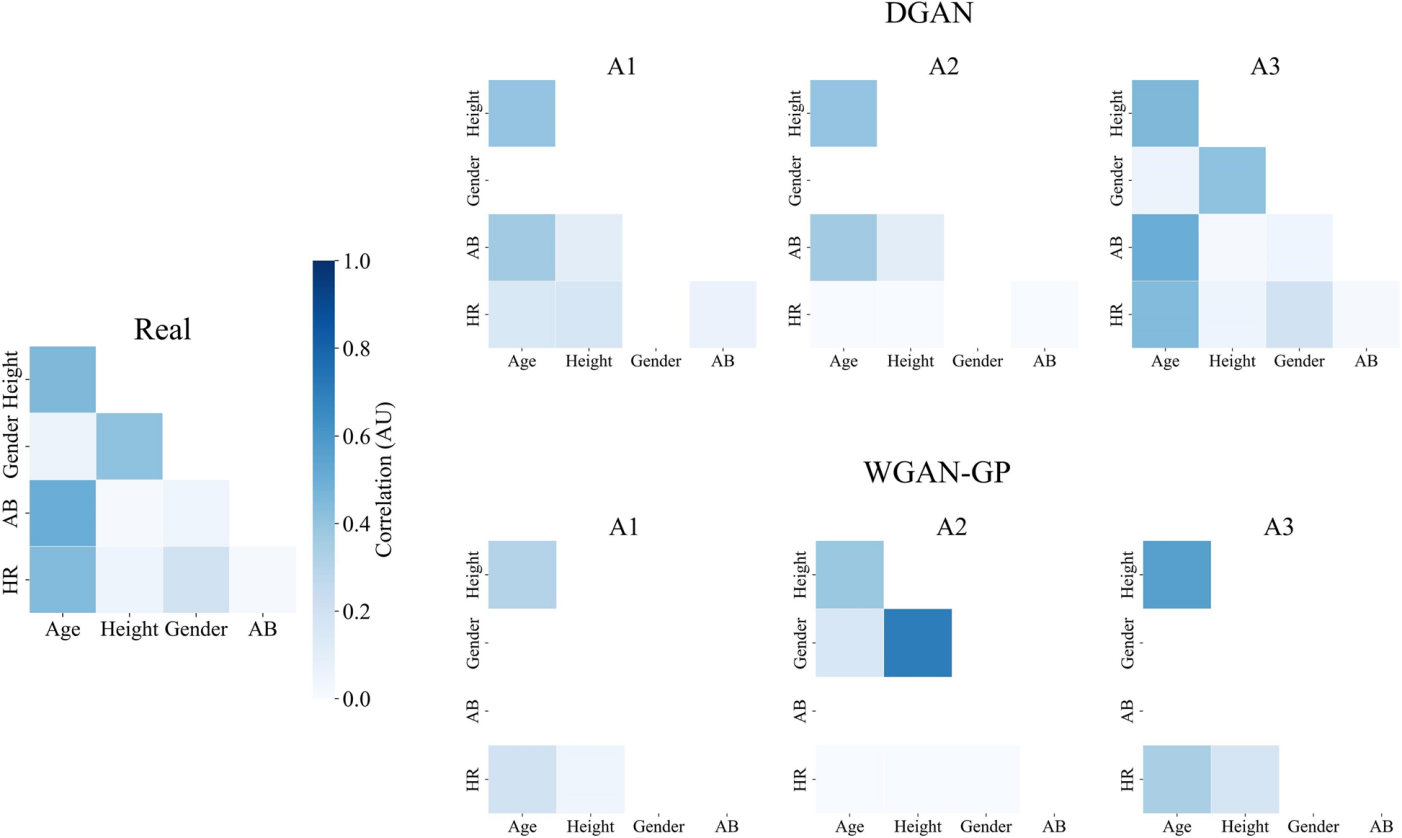
Multivariate correlations of the real (left) and synthetic PMData datasets. The upper row shows the data generated with the DGAN, while the lower row shows the data generated with the WGAN-GP. The three columns on the right refer to the different approaches followed during the synthesis. HR: Heart Rate. Note that blank values appear for the features with a standard deviation equal to zero, which lead to a null value in correlation calculations

As for the third multivariate analysis, Fig. 5 shows the correlations that were calculated for the PMData dataset. The A3 approach for the DGAN_P_ was the only model that managed to produce the correlations of the original dataset as the rest of the models missed to capture the ones that involved binary variables. The A1 approach was the best for the WGAN-GP_P_ model, but the similarity metrics in Table 4 show poor results.

The DLA analysis on the TMET dataset proved to be similar for every approach, resulting in values near 0.9 in every experiment. Regarding the hypotension subset, the best results were achieved with the first approach (A1), as the predictions were closer to 0.5. The best-performing WGAN-GP_T_ model on the DLA assessment was obtained with the A3 approach, even if closely followed by the other methods. Moreover, the assessment on the WGAN-GP_M_ models show that the lowest classificability of the samples is achieved when following the A1 approach. There was no difference in the DLA analysis for the PMData dataset as the samples were properly distinguished by the classifiers in every case.

To finish with the resemblance assessment, the autocorrelation analysis was performed considering the time-dependent variables in each dataset. As evaluating the A1 approach would not make sense (both the generated and the original data contain the same real time series), these values were set to use them as baselines for the other two methods. The A2 approach was the method that best emulated the autocorrelation metrics in A1 for the hypotension subset, the PMData dataset, and the WGAN-GP_T_ model, while A3 was the best-performing one for DGAN_T_.

The utility metrics derived from the DGAN_T_ models show the most useful data was generated using the A3 approach, while A1 was the worst-performing one, even if the statistical significance was not proven as it can be checked in Table 3. On the other hand, DGAN_M_ models demonstrate the opposite for the hypotension subset, the first approach being the best-performing one (*p* > 0.05). Concerning the WGAN-GP models, the ones trained with the TMET dataset show the best approach is also A3, even if not far from the other two methods. The WGAN-GP_M_ combination performed the best using the A2 approach. No statistical test associated with these model and dataset combinations resulted in statistical significance. Regarding the PMData dataset, the A1 approach performed best in the DGAN case (*p* < 0.05), as well as for the WGAN-GP_P_ (*p* < 0.05). In Table 5 the values obtained for the TSTR evaluation can be compared.

**Table 5:** Utility metric results

Generation Method	Model^a^	TSTR metric
**A1**	DGAN_T_	0.802 ± 0.033
	WGAN-GP_T_	0.880 ± 0.002
	DGAN_M_	**0.931 ± 0.064**
	WGAN-GP_M_	0.909 ± 0.065
	DGAN_P_	**0.983 ± 0.005**
	WGAN-GP_P_	**0.986 ± 0.002**
**A2**	DGAN_T_	0.900 ± 0.589
	WGAN-GP_T_	0.837 ± 0.109
	DGAN_M_	0.869 ± 0.078
	WGAN-GP_M_	**0.924 ± 0.049**
	DGAN_P_	0.906 ± 0.083
	WGAN-GP_P_	0.919 ± 0.000
**A3**	DGAN_T_	**0.919 ± 0.167**
	WGAN-GP_T_	**0.900 ± 0.156**
	DGAN_M_	0.893 ± 0.079
	WGAN-GP_M_	0.758 ± 0.125
	DGAN_P_	0.957 ± 0.016
	WGAN-GP_P_	0.778 ± 0.120

Values in bold highlight the best-performing approach per trained model and metric

*TSTR* Train on Synthetic and Test on Real

^a^The subscripts on the model column refer to the dataset used for the training step

**Fig. 6:**
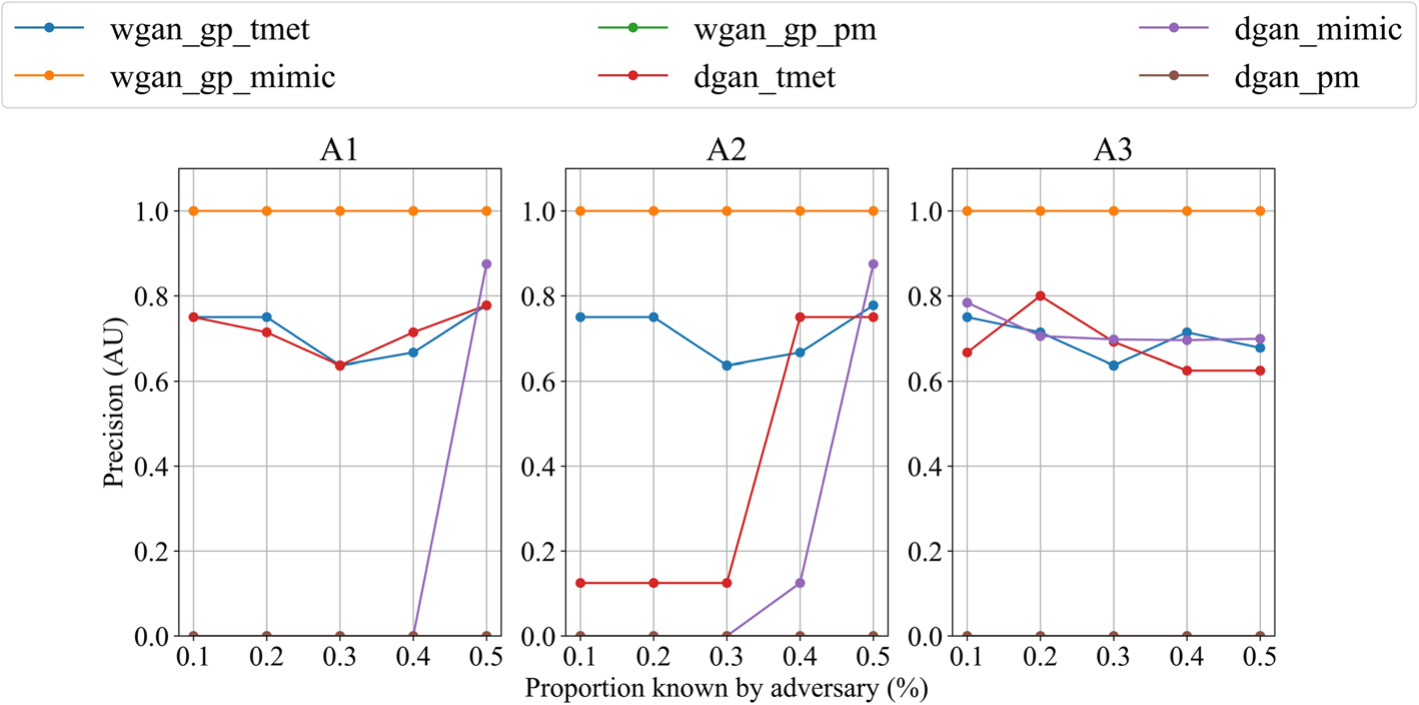
Membership Inference Attack (MIA) precision results. Each subplot represents the approach that was followed to synthesize the data, and each curve represents the pseudo-cross-validated mean precision of a MIA. AU: Arbitrary Unit

The last results to be presented are those of the privacy assessment. Regarding the WGAN-GP models, both datasets yielded similar values among approaches: the WGAN-GP_T_ resulting in precision values around 0.7 with a slightly better outcome for A3 in the highest proportions known by the attacker, and the WGAN-GP_M_ not being able to counter the MIA in any scenario. For the WGAN-GP_P_ models, all the approaches resulted in private data as a zero precision was achieved during the MIA simulations. With respect to DGAN_T_, the best results were achieved with A2 and for low proportions of the dataset known by the adversary (0.1–0.3). For higher proportions (0.4–0.5), comparable results were obtained with every tested approach. The DGAN_M_ model in A1 and A2 outperformed the A3 approach for the lowest proportions (0.1–0.3) with zero precision on the MIA setup, even if for the highest proportions the precision deteriorated significantly. Last, the DGAN models trained with the PMData dataset resulted in private data again as the precision was zero for all the approaches. The MIA simulation results are graphically represented in Fig. 6.

## Discussion

### Resemblance assessment

The resemblance results presented on the evaluation section were found to follow a pattern dependent on the initial characteristics of the dataset to be synthesized. Specifically, the time-series-to-metadata ratio, which refers to the number of longitudinal variables with respect to the static ones in a dataset, was found to predetermine resemblance metrics, as well as the length of the time series, as these are determinant on the data quantity that remains real in the A1 approach. In fact, the time-series-to-metadata ratio in the TMET dataset was 6:5, from which it can be inferred that 54.5% of the dataset variables were time series, while for the hypotension subset a ratio of 6:2 constituted the 75% of the dataset variables being longitudinal, and for the PMData dataset the ratio was 1:4. Considering this fact, A1 seems to be favoured when higher ratios and/or longer series are used, since a higher percentage of the generated SD remains real, achieving improved resemblance metrics. In principle, approaches A2 and A3 should not depend on this ratio as both metadata and time series are synthesized in these approaches.

Regarding statistical analysis of results on the resemblance metrics, most of correlation similarity checks, autocorrelation measurements and F-score metrics resulted in statistically significant differences. DLA was demonstrated to be statistically significant just for the MIMIC dataset, while no difference was found for TMET and PMData ones.

### Utility assessment

Regarding the utility aspect of the generated data, the approach A3 was the best performing one for the TMET data, while approach A1 was better for PMData and DGAN_M_. This could lead to a similar rationale as discussed in the resemblance section, where a larger quantity of real data could potentially enhance the outcomes in the resultant datasets.

As for the significance of the performed comparisons, neither the models trained with the TMET dataset, nor the DGAN_M_ model were found to be statistically significant. In contrast, both the DGAN and the WGAN-GP trained with the PMData dataset and the WGAN-GP_M_ model were found to be different to a great extent.

### Privacy assessment

The MIA setup used for evaluation in this study is stringent, but no standardized time-series-specific MIA setup was found in the literature either. Future work in the area of privacy assessment will comprise research and incorporation of different attacks and thresholds to create potential scenarios that encompass a broader range of possibilities.

The WGAN-GP model was found to be susceptible to mode-collapse [26], which might explain the precision achieved by the adversary when attacking the WGAN-GP_M_ generated data. Finding no significant differences in the WGAN-GP_T_ suggests no approach is better than the other in terms of privacy preservation.

Attacks to the data generated with DGAN_T_ suggest the most privacy-preserving approach is A2, which is consistent with the utility metrics that were obtained with it (best utility is achieved with A3). Contrary to our initial hypothesis, the privacy assessment on DGAN_M_ resulted in approaches A1 and A2 outperforming A3 when using the DGAN model, which might relate to how the MIA parameters were configured.

Regarding the MIA simulations performed with the PMData dataset, the low number of subjects in the original dataset may have conditioned the results. A lower number of subjects decreases the possibility to find a potential match between records as fewer calculations are performed.

### Contributions and limitations

The main contribution of this work is the detailed definition and systematic comparison of the three methods for generating time series together with metadata in terms of different evaluation dimensions, presenting them in a common framework and substantially extending analyses previously published by the authors in [12, 13].

The first limitation of the study is related to the fact that no standardized metrics can be found in the literature to evaluate longitudinal synthetic datasets. Even if utility and resemblance metrics can be adapted easily to this use case, it was difficult to objectively evaluate the privacy of the data considering the different thresholds and parameters that were introduced during the assessment, as well as the differences between the datasets that were used. Also, even if the longitudinal variables were jointly evaluated with the metadata, autocorrelation was the only time-series-specific metric that was calculated. For the future, a deeper research on this aspect is planned by analysing time series features such as central tendency (e.g. mean, mode and median), variability metrics (e.g. range and variance), trend metrics (e.g. slope), or seasonality metrics (e.g. seasonal index and cycle).

Moreover, the generated SD did not overcome any clinical validation and, therefore, the utility of the data for real scenarios or use cases was evaluated just by simulating possible downstream applications with the data as it is. Conclusions drawn from analyses derived from the SD should be checked and discussed by clinical experts to provide enough evidence for using SD in real scenarios or use cases.

## Conclusion

In this research, a comparative assessment of three different STSG methods was conducted, evaluating three key dimensions of SD: resemblance (1), utility (2), and privacy (3). Although it may not be entirely decisive for setting a gold standard, the research was useful to state that the A2 approach performed worst overall, and therefore, approaches A1 and A3 should be prioritized for any use case.

Depending on the dataset characteristics, the use case, and the risk to be assumed by an end-user, the findings of this analyses point the user in the direction of implementing either A1 or A3 with regard to resemblance and utility metrics. This recommendation should be analyzed more cautiously before generalazing it, as further metric calculations may prompt a shift in direction. The privacy assessment is warranted as a main future work line, including the evaluation of datasets with different characteristics (e.g. low subject quantity), since it is pivotal for SD sharing or publication.

## Data Availability

The hypotension data that supports the conclusion of this study was taken from the MIMIC-III v1.4 clinical database and it is available in *Physionet* for credentialed users who sign the DUA (Data Use Agreement), https://physionet.org/content/mimiciii/1.4/. The Treadmill Maximal Effort Exercise Test (TMET) data that supports the conclusion of this study is also available via *Physionet* for users conforming to the terms of the specified license, https://physionet.org/content/treadmill-exercise-cardioresp/1.0.1/.

## Abbreviations

AU: Arbitrary Unit
AUROC: Area Under the Receiver Operating Characteristics curve
DGAN: DöppelGANger
DLA: Data Labelling Analysis
DT: Decision Trees
GAN: Generative Adversarial Network
GCP: Google Cloud Platform
GCS: Glasgow Coma Scale
GDPR: General Data Protection Regulation
GET: Graded Exercise Test
HIPAA: Health Insurance Portability and Accountability Act
HR: Heart Rate
KNN: K-Nearest Neighbors
MAP: Mean Arterial Pressure
MIMIC: Medical Information Mart for Intensive Care
MLP: Multi-Layer Perceptron
NSD: Norwegian Centre for Research Data
PII: Personally Identifiable Information
RD: Real Data
RF: Randon Forest
RNN: Recurrent Neural Network
RR: Respiratory Rate
SD: Synthetic Data
SDG: Synthetic Data Generation
SL: Significance Level
STSG: Synthetic Time Series Generation
SVM: Support Vector Machines
TMET: Treadmill Maximal Exercise Test
TRTR: Train on Real and Test on Real
TSTR: Train on Synthetic and Train on Real
VE: Exhaled Volume
WGAN-GP: Wasserstein Generative Adversarial Network with Gradient Penalty
